# New combinations and updated descriptions in *Podagrostis* (Agrostidinae, Poaceae) from the Neotropics and Mexico

**DOI:** 10.3897/phytokeys.148.50042

**Published:** 2020-05-26

**Authors:** Steven P. Sylvester, Paul M. Peterson, Konstantin Romaschenko, William J. Bravo-Pedraza, Lia E. Cuta-Alarcon, Robert J. Soreng

**Affiliations:** 1 College of Biology and the Environment, Nanjing Forestry University, Long Pan Road No. 159, Nanjing, 210037, China Nanjing Forestry University Nanjing China; 2 Royal Botanic Gardens, Kew, Richmond, Surrey, TW9 3AE, UK Royal Botanic Gardens Kew United Kingdom; 3 Department of Botany, National Museum of Natural History, Smithsonian Institution, Washington DC 20560, USA National Museum of Natural History, Smithsonian Institution Washington United States of America; 4 Grupo Sistemática Biológica, Herbario UPTC, Escuela de Biología, Facultad de Ciencias, Universidad Pedagógica y Tecnológica de Colombia, Avenida Central del Norte 39-115, Tunja-Boyacá, Colombia Universidad Pedagógica y Tecnológica de Colombia Tunja-Boyacá Colombia

**Keywords:** *Agrostis*, Andes, Central America, Colombia, Ecuador, páramo, Peru, Venezuela

## Abstract

Based on morphological study and corroborated by unpublished molecular phylogenetic analyses, five grass species of high-mountain grasslands in Mexico, Central and South America, *Agrostisbacillata*, *A.exserta*, *A.liebmannii*, *A.rosei*, and *A.trichodes*, are transferred to *Podagrostis* and bring the number of species of this genus recognized in the New World to ten. The name *Aperaliebmannii* is lectotypified and epitypified. We provide an updated genus description for *Podagrostis*, and updated species descriptions, images, and notes on the new combinations. The diagnostic characteristics differentiating *Podagrostis* from *Agrostis* are: a) palea that reaches from (2/3) ¾ to almost the apex of the lemma; b) florets that usually almost equal the length of the glumes or are at least ¾ the length of the glumes; c) rachilla extension present and emerging from under the base of the palea as a slender short stub (rudimentary or up to 1.4 mm long, sometimes obscure in most florets in *P.rosei*), smooth or scaberulous, glabrous or distally pilulose (hairs < 0.3 mm long); d) lemmas usually awnless, sometimes with a short straight awn 0.2–0.6 mm long, inserted medially or in the upper 1/3 of the lemma, not surpassing the glumes (awn well-developed, straight or geniculate and inserted in lower 1/3 of lemma, not or briefly surpassing glumes in *P.rosei*). We include a generic key to distinguish the species of *Podagrostis* from other similar genera in Latin America and a key to distinguish the species of *Podagrostis* now accepted as occurring in these areas.

## Abstract (pt)

Basado en estudios morfológicos y corroborado por datos no publicados de análisis filogenéticos, las cinco gramíneas de alta montaña de los pastizales en México, Centroamérica y Sur América *Agrostisbacillata*, *A.exserta*, *A.liebmannii*, *A.rosei*, y *A.trichodes*, son transferidos a *Podagrostis* incrementando a diez el número de especies en el nuevo mundo de este reconocido género. El nombre de *Aperaliebmannii* también es lectotipificado y epitipicado. Proporcionamos una descripción actualizada del género *Podagrostis*, y descripciones actualizadas de las especies, imágenes y comentarios sobre las nuevas combinaciones. Las características diagnósticas que diferencian a *Podagrostis* de *Agrostis* son: a) pálea de 2/3-¾ hasta casi el ápice de la lemma; b) espiguillas, generalmente casi iguales en longitud a las glumas o de al menos ¾ la longitud de las glumas; c) extensión de la raquilla presente y emergiendo desde debajo de la base de la pálea como un trozo corto y delgado (desde rudimentario hasta de 1.4 mm de largo, a veces oscuro en la mayoría de los flósculos en *P.rosei*), liso o escabérulo, glabro o piloso distalmente (pelos < 0.3 mm de largo); d) lemmas generalmente sin arista, o a veces con arista corta y recta de 0.2–0.6 mm de largo, insertada medialmente o en el tercio superior de la lemma, sin sobrepasar las glumas (arista bien desarrollada, recta o geniculada e insertada en la parte inferior 1/3 de lemma, no o superando brevemente las glumas en *P.rosei*). Incluimos una clave para distinguir *Podagrostis* de otros géneros similares presentes en América Latina y una clave para distinguir las especies de *Podagrostis* ahora aceptadas por estar presentes en estas áreas.

## Introduction

*Agrostis* L. and *Podagrostis* (Griseb.) Scribn. & Merr. both belong to the tribe Poeae R.Br., subtribe Agrostidinae Fr. s.s. (Soreng et al. 2017), and share numerous characteristics, including paniculate, single-flowered laterally-compressed spikelets that disarticulate above the glumes. Delimitation of these two genera has been complicated by a lack of surety of the morphological discontinuities, with Podagrostis originally described as a section of Agrostis, and some accounts treating the former as a taxonomic synonym of the latter (e.g. Clayton and Renvoize 1986; Pohl and Davidse 1994). The distinction of *Podagrostis* was corroborated by molecular phylogenetic research, with plastid analyses finding *Podagrostisaequivalvis* (Trin.) Scribn. & Merr. to be placed with *Calamagrostisbolanderi* Thurb. in a maximally supported basal lineage sister to a clade comprising *Agrostis*, *Polypogon* Desf., and four Chinese species of “*Deyeuxia*” Clarion ex P.Beauv. plus *Agrostisrosei* Scribn. & Merr. from Mexico that did not resolve with *Calamagrostis* Adans. sensu Soreng et al. (2017) (Saarela et al. 2017). Current treatments recognize five species of *Podagrostis*, three from North America (Roché et al. 2007) and two from South America (Rúgolo de Agrasar 2012; Sylvester et al. 2019a).

Morphologically, these taxa of *Podagrostis* differ from currently circumscribed *Agrostis* taxa in the combination of a well-developed, relatively long palea that reaches from (2/3) ¾ to almost the apex of the lemma, florets that usually almost equal the length of the glumes or which at least reach past ¾ the length of the glumes, the presence of a slender rachilla prolongation in all or most spikelets that is smooth or scaberulous, with hairs lacking or distally pilulose (hairs < 0.3 mm long), and lemmas usually awnless or with a short straight awn 0.2–0.6 mm long, inserted medially or in the upper 1/3 of the lemma, not surpassing the glumes. These characters are also found in the species *Agrostisliebmannii* (E. Fourn.) Hitchc. and *A.rosei* (awn well-developed, straight or geniculate and inserted in lower 1/3 of lemma, usually not surpassing glumes in *A.rosei*) from mountains of central and southern Mexico, *A.exserta* Swallen from high-elevation grasslands of Guatemala, and *A.bacillata* Hack. and *A.trichodes* (Kunth) Roem. & Schult. from high-elevation páramo grasslands of Costa Rica and Panama, or northwest South America, respectively. The possible affinity of some of these species to *Podagrostis*, based on the presence of a rachilla prolongation, has been suggested (Pohl and Davidse 1994; Briceño 2010), and the placement of *A.rosei* in the plastid tree in Saarela et al. (2017) provided molecular support for this idea. All other *Agrostis* taxa with well-developed paleas in Mexico, Central America and northwest South America, with which these species could possibly be confused (because their paleas sometimes exceed 2/3 the length of their lemmas), are exotics introduced from the Old World (i.e. *A.alba* L.; *A.capillaris* L.; *A.castellana* Boiss. & Reut.; *A.gigantea* Roth; *A.stolonifera* L.) that lack a rachilla prolongation and have florets that are 1/3–3/4 the length of the glumes, and paleas ranging from 2/5 to 2/3 (rarely reaching to ¾) the length of the lemmas. The other native *Agrostis* species from the region we are discussing have shorter, often rudimentary or absent paleas.

In a large unpublished molecular DNA sequence study using three plastid gene regions (*rpl32-trnL* spacer, *rps16-trnK* spacer, and *rps16* intron), Romaschenko et al. (pers. comm.) found that *A.bacillata* and *A.rosei* were closely related to other taxa of North American *Podagrostis* [*P.aequivalvis*; *P.humilis* (Vasey) Björkman; *P.thurberiana* (Hitchc.) Hultén]. These form a clade sister to *Agrostis* and *Polypogon*. Saarela et al. (2017) also reported a similar result for *A.rosei* in their plastid tree, which found the taxon to be part of a well-supported clade with four Chinese species of *Deyeuxia*, and *Calamagrostisbolanderi* + *Podagrostisaequivalvis*. Some of the aforementioned Chinese *Deyeuxia*’s and *C.bolanderi* from California remain to be sampled using the same markers used in this study to clarify their position. Although *C.bolanderi*’s placement in a strongly supported lineage with *Podagrostisaequivalvis* (Saarela et al. 2017) provides support for its transferal to *Podagrostis*, it may represent a separate hybrid between *Podagrostis* and *Calamagrostis*.

Nuclear ribosomal internal transcribed spacer DNA sequences of all these species resolved within *Calamagrostis* sensu Peterson et al. (2019), which is also congruent with placement of *P.aequivalvis* in the ITS and ITS+ETS tree and of *A.rosei* in the ITS tree in Saarela et al. (2017). Saarela et al. (2017) also mentioned *A.rosei* to be placed outside of two strongly supported clades that include all sampled species of *Agrostis*, *Polypogon* and one species of *Lachnagrostis* in their more well-resolved, albeit more poorly sampled, ITS-ETS tree (Saarela et al. 2017: 65, not shown in Saarela et al. 2017: fig. 7), further confirming the distinctiveness of this taxon. The contrasting nrDNA and plastid placements indicate *Podagrostis* is reticulate in origin between *Calamagrostis* and a sister lineage to *Agrostis* plus *Polypogon*, and may yet extend to Asian elements.

The molecular results support our transfer of these and other morphologically similar *Agrostis* species, *A.exserta*, *A.liebmannii*, and *A.trichodes*, to *Podagrostis*. We propose five new combinations, *Podagrostisbacillata*, *P.exserta*, *P.liebmannii*, *P.rosei*, and *P.trichodes*, and provide images, up-to-date descriptions, and notes for each species. We also present a key to distinguish *Podagrostis* from similar genera in the Neotropics and Mexico, and a key to distinguish *Podagrostis* species from these areas.

## Materials and methods

Accepted species follow Soreng et al. (2003 and onwards). Herbarium acronyms follow Index Herbariorum (Thiers, continuously updated). In this treatment, unless otherwise stated, glabrous means without pubescence (in the sense of slender, relatively soft hairs). Smooth indicates no prickle-hairs with broad bases and/or hooked or pointed apices (i.e., pubescence can occur on a smooth surface, and a rough or scabrous surface can be glabrous). Specimens in the herbaria COL, FMB, K, UPTC, and US were reviewed for this study. Beyond types (some only seen in images), only material from herbaria where specimens have been checked and verified by the authors are cited. Parts of the generic key related to taxa of previously circumscribed *Calamagrostis* s.l. were adapted from Peterson et al. (2019).

## Taxonomic treatment

### Key to distinguish *Podagrostis* from other Latin American, one-flowered genera of tribe Poeae with both glumes as long or longer than the floret

**Table:** 

1	Spikelets disarticulating below the glumes, the glumes and floret, and often part of the pedicel, falling together as a unit; glumes awned or muticous	**2**
–	Spikelets disarticulating above the glumes, the glumes remaining on the inflorescence after the florets have fallen; glumes acute to acuminate, not awned	**5**
2	Palea < ½ the length of the lemma; glume apices lanceolate or lanceolate-subulate, muticous, mucronulate or awned	***Chaetotropis* Kunth**
–	Palea equaling or subequaling the lemma; glume apices obtuse or bilobed (*Polypogon*) or muticous (*Cinna*), awned or unawned	**3**
3	Panicles condensed, generally spikelike; glumes much longer than the lemma; glume apices obtuse or bilobed, awned or unawned; rachilla extension absent	***Polypogon* Desf.**
–	Panicles open to loosely contracted, somewhat lax; glumes as long as the lemma; glume apices muticous, unawned; rachilla extension usually present as a short glabrous stub	**4**
4	Lemma with a subapical awn 5–14.5 mm long; glumes coriaceous, rigid, hispid or scabrous throughout; anthers 3	***Limnodea* L.H. Dewey**
–	Lemma unawned or with a subapical awn < 1 mm long; glumes membranous, flexible, glabrous, scabrous only on the keel and sometimes lateral veins; anthers 1 or 2	***Cinna* L.**
5	Rachilla extension absent; palea of varying length; callus glabrous or shortly pubescent with hairs to 0.5 mm long	**6**
–	Rachilla extension present (cases where it is sometimes rudimentary key both ways), of varying lengths (sometimes very short, and requiring the base of the palea be checked closely to distinguish the structure from hairs) smooth or scaberulous, glabrous or pilulose to pilose, NB. (absent in *Calamagrostisllanganatensis* Laegaard but then callus hairs reaching from ½ to ¾ the length of the lemma); palea well-developed, generally > 2/3 the length of the lemma; callus glabrous, shortly pubescent, or notably pubescent with long hairs	**9**
6	Floret stipitate (lowermost rachilla internode distinctly elongated between the glumes and the floret), stipe 0.2–4 mm long, cylindrical, dilated towards its apex (can be seen at the base of the glumes after the floret has fallen); spikelets > 4 mm long; palea well-developed, subequaling the lemma	***Deschampsia* P. Beauv.** (in part)
–	Floret sessile (lowermost rachilla internode not, or not noticeably, prolonged between the glumes and the floret), stipe less than 0.2 mm long; spikelets commonly < 4 mm long; palea of varying length, well-developed and subequaling or equaling the lemma to minute/absent	**7**
7	Lemma apex terminating in 4 scabrous setae; lemma surface often pilose; lemma with a well-developed geniculate awn inserted in the lower 1/3 and surpassing the glumes; calluses pilulose; caryopsis thin, with liquid endosperm	***Bromidium* Nees & Meyen** (likely a synonym of *Agrostis*; Nogueira da Silva et al. 2020)
–	Lemma apex entire or finely dentate with short teeth at the end of each lateral vein; lemma surface glabrous (rarely with a few hairs in *Agrostiscastellana* L.); lemmas muticous, with a short straight awn 0.2–1 mm long, or with a long geniculate and twisted awn to 6+ mm long, inserted basally, medially or in the upper half of the lemma, not surpassing to greatly surpassing the glumes; calluses usually glabrous or with hairs restricted to lateral lines continuous with the basal lemma margins; caryopsis usually rounded, with hardened endosperm	**8**
8	Floret equaling or subequaling the glumes, sometimes slightly shorter but reaching past ¾ the length of the glumes, usually with a short rachilla prolongation emerging behind the palea (sometimes absent in many florets of *P.rosei* so check many spikelets); paleas well-developed, usually reaching from (2/3) ¾ to almost the apex of the lemma; panicles 1–6.5(–11) cm long (up to 18 cm in the Mexican species *P.liebmannii* and *P.rosei*); lemmas muticous or with a short straight awn 0.2–0.6 mm long, inserted medially or in the upper half of the lemma, not surpassing the glumes (awn well-developed, 1.6–2 mm long, inserted in lower 1/3 of lemma, straight or geniculate and usually not surpassing glumes in *P.rosei*)	***Podagrostis* (Griseb.) Scribn. & Merr.** (in part)
–	Floret notably shorter than the glumes, usually 1/3–3/4 the length of the glumes, rarely longer, without a trace of a rachilla prolongation; paleas well-developed, poorly-developed, or absent, when well-developed reaching from ½–¾ the length of the lemma; panicles often > 5 cm long; lemmas muticous, with a short straight awn 0.2–1 mm long, or with a long geniculate and twisted awn to 6+ mm long, inserted basally, medially or in the upper half of the lemma, not surpassing to greatly surpassing the glumes	***Agrostis* L.**
9	Lemmas densely pubescent, with rigid and abundant hairs that cover the lemma veins	**10**
–	Lemmas glabrous or pilose only at the basal margins, with veins evident at least in the upper part	**11**
10	Culms fragile at maturity, inflorescence often disarticulating with age; panicles generally open, with divaricate branching	***Lachnagrostis* Trin.**
–	Culms slender or stout, not disarticulating with age; panicles contracted or open, without divaricate branching (in species with hairy lemmas)	***Peyritschia* E. Fourn.** (in part)
11	Floret stipitate (lowermost rachilla internode distinctly elongated between the glumes and the floret), stipe 0.2–4 mm long, cylindrical, dilated towards its apex (can be seen at the base of the glumes after the floret has fallen); callus and rachilla glabrous (rarely with a few short callus hairs); ligules slightly to strongly decurrent, usually elongated, 4–20 mm long, acuminate, smooth or nearly so, glabrous, entire or laterally cleft	***Deschampsia* P. Beauv.** (in part)
–	Floret sessile or subsessile (lowermost rachilla internode not, or not noticeably, prolonged between the glumes and the floret), stipe < 0.2 mm long; callus and rachilla glabrous or pubescent; ligules decurrent or not, 0.2–10 (–15) mm long, commonly less than 4 mm long (lateral lobes often exceeding the central part), often scabrous or pubescent, commonly truncate to obtuse	**12**
12	Lemmas unawned or with a short straight awn, usually < 0.5 mm long, inserted in the upper half of the lemma, not or barely exceeding the glumes (awn well-developed, 1.6–2 mm long, inserted in lower 1/3 of lemma, straight or geniculate and usually not surpassing glumes in *Podagrostisrosei*, but then callus glabrous, rachilla very short, < 0.3 mm long, glabrous, plants from Mexico)	**13**
–	Lemmas with a well-developed usually geniculate and twisted awn (sometimes flexuose in e.g. *Apera*), > 1 mm long, inserted in the lower or upper half of the lemma, clearly exceeding the glumes	**17**
13	Callus and rachilla glabrous [*Podagrostissesquiflora* (E. Desv.) Parodi ex Nicora often with short hairs emerging from only the rachilla apex and the basal side-ridges of the callus]	**14**
–	Callus and rachilla hairy (NB. Rachilla absent in *Calamagrostisllanganatensis*)	**15**
14	Anthers 3 in number, 0.4–1 mm long (to 2.2 mm long in *P.colombiana* Sylvester & Soreng); spikelets < 4 mm long (sometimes to 4.2 mm long in *P.colombiana*); panicles 1–6.5(–11) cm long in taxa from Central and South America, 7–17 cm long in *P.liebmannii* and *P.rosei* from Mexico; palea keels smooth	***Podagrostis* (Griseb.) Scribn. & Merr.** (in part)
–	Anthers 3 or 1 in number, 1.2–3 mm long; spikelets 3.5–6+ mm long; panicles usually >11 cm long; palea keels often scabrous, at least in part, sometimes smooth throughout (e.g. *C.carchiensis* Lægaard)	***Calamagrostis* Adans.** (in part)
15	Rachilla absent	***Calamagrostisllanganatensis* Laegaard**
–	Rachilla present	**16**
16	Florets with a short stipe (± 0.15 mm long) between the upper glume and the callus of the floret; both glumes 3-veined; caryopsis hard, hilum linear ± 1/3 the grain in length; plants from the páramo in Ecuador	***Laegaardia* P.M. Peterson, Soreng, Romasch. & Barberá**
–	Florets sessile; lower glumes 1-veined, upper glumes 1- or 3-veined; caryopses hard to pasty, hilum oval to punctiform < 1/4 the grain in length; plants from various locations, including Ecuadorian páramos	***Cinnagrostis* Griseb.** (in part)
17	Anthers 2, or 3 in plants from Mexico and Guatemala; lemma body strongly 5-veined, often puberulent in part	***Peyritschia* E. Fourn.** (in part)
–	Anthers 1 or 3, if 3 then plants not from Mexico (*Calamagrostisguatemalensis* Hitchc. from Guatemala); lemma body variously veined, glabrous, sometimes scabrous	**18**
18	Panicles open, diffuse; rachilla extension nearly as long as lemma, densely and evenly hairy with hairs 0.5–1.2 mm long; lemmas awned from middle, the awns 2–7.2 mm long, basally twisted, geniculate; callus hairs 0.2–0.8 mm long; leaf blades readily to tardily disarticulating from collars in age (tending to form a J at base after falling), involute, sometimes sinuous; caryopsis hard, hilum linear ± 1/3 the grain in length; lower glumes 1-veined, upper glumes 3-veined and sometimes with 1 or 2 cross-veins between them; plants from páramos of Colombia, Ecuador, and Venezuela	***Paramochloa* P.M. Peterson, Soreng, Romasch. & Barberá**
–	Panicles open (infrequently diffuse) or contracted; rachilla extension mostly less than ¾ the lemma in length, variously hairy, the hairs 0.5–4 mm long, sometimes reduced or absent proximally; lemmas awned from near base to upper 1/3, the awns 1–10 mm or more long, straight, sinuous or geniculate; callus hairs absent to 4 mm long; leaf blades not disarticulating from collars in age, flat, folded or involute, not sinuous; caryopsis soft or hard; hilum elliptical, oval, round to punctiform 1/5–1/3 the grain in length; lower glumes 1–3-veined without cross-veins between them; plants of various habitats, from Mexico to Tierra del Fuego, Argentina	**19**
19	Caryopses hard, distinctly sulcate, hilum 1/6–1/3 the grain in length; lemmatal awns strait or slightly bent, readily distinguished from callus hairs, inserted from near base to middle, not or slightly exceeding the lemma apex; callus hairs 0.1–3 mm long 1/10–3/4 as long as the lemma in length; rachilla glabrous, or sparsely to densely hairy, hairs not reaching lemma apex; panicles contracted; anthers 1 or 3; lodicules entire and lanceolate, sometimes with an isolated lateral lobe, glabrous	***Calamagrostis* Adans.** (in part)
–	Caryopses soft (liquid or semi-soft) or hard, sulcate (often shallowly) or not, hilum 1/6–1/4 the grain in length (often obscure in species with lipid); lemmatal awns sometimes indistinguishable from callus hairs, straight, sinuous or geniculate, capillary or stout, inserted from base to upper 1/3, usually exceeding the lemma and often exserted from the glumes; callus hairs shorter to exceeding the lemma in length; rachilla hairy, hairs often reaching 3/4 to exceeding the lemma length; panicles contracted or open; anthers 3; lodicules apically bilobate or bidentate, infrequently entire and lanceolate, apical margin sometimes ciliolate or ciliate	***Cinnagrostis* Griseb.** (in part)

#### Podagrostis

Taxon classificationPlantaePoalesPoaceae

(Griseb.) Scribn. & Merr. Contr. U.S. Natl. Herb. 13(3): 58. 1910.

E5C88B7B-5B37-5AC1-983D-2A570BB0312A

Agrostis sect. Podagrostis Griseb. Fl. Ross. 4(13): 436. 1852.

##### Type.

Agrostiscaninavar.aequivalvis Trin. (lectotype designated by: Hitchcock 1920: 127).

##### Description.

***Perennials***, loosely to densely tufted, sometimes forming small tussocks, sometimes subrhizomatous (North American, including Mexico, and Austral South American taxa). ***Culms*** 5–90 cm tall, slender. ***Tillers*** either extra- or intravaginal. ***Leaves; ligules*** 0.2–5.5 mm long, hyaline, glabrous, smooth or lightly scabrous, apices truncate, obtuse, acute or acuminate, entire to lacerate; ***blades*** involute, folded, or flat. ***Inflorescence*** 1–12 cm long, a panicle, lax and open to loosely to moderately densely contracted; ***panicle branches and pedicels*** glabrous, often smooth or infrequently scaberulous. ***Spikelets*** 1–4.2 mm long, 1-flowered, disarticulating above the glumes, weakly laterally compressed; ***glumes*** equal or subequal, the lower often longer than the upper, equaling or subequaling the length of the floret or slightly longer, persisting on the plant after the florets have fallen or sometimes readily caducous, glabrous, keel smooth or usually scabrous at least distally, lateral veins smooth or slightly scabrous distally, surfaces usually smooth, less often scabrous; lower glume 1- or 3-veined; upper glume 1- (2-) or 3-veined; ***floret*** 1 in number, sessile, subequaling to equaling the apex of the glumes; ***lemmas*** membranaceous, often slightly thicker than the glumes, dorsally rounded, 3- or 5-veined, lateral veins not evident to distinct, glabrous, smooth or scabrous, apex muticous or with a short straight awn 0.2–0.6 mm long, inserted medially or in the upper 1/3 of the lemma, not surpassing the glumes, (awn well-developed, 1.6–2 mm long, inserted in lower 1/3 of lemma, straight or geniculate and usually not surpassing glumes in *P.rosei*); ***paleas*** well-developed, reaching from (2/3) ¾ to subequaling the lemma, keels obscure to distinct, glabrous, smooth; ***calluses*** rounded, blunt, usually glabrous, or with two short lateral tufts of hairs to 0.5 mm long in some species, abaxially smooth; ***rachilla*** prolongation present, slender, varying from rudimentary to 2/3 the floret in length (obscure or absent in many *P.rosei* spikelets), glabrous or sometimes with short strict hairs to 0.3 mm long emerging only distally, smooth or scaberulous. ***Flowers*** perfect; ***lodicules*** 2 in number; ***anthers*** 3 in number, 0.3–1.6 mm long (–2.2 mm long in *P.colombiana*); ***ovaries*** glabrous. ***Caryopses*** slightly shorter to equaling the lemmas, concealed at maturity, subterete to fusiform, hardened, sulcus distinct; hilum punctiform to narrowly ovoid; embryo c. ¼–1/3 length of the caryopsis; endosperm solid (information on caryopses taken from Harvey 2007, Rúgolo de Agrasar 2012 and *P.trichodes* specimens). 2*n* = 14 (in *P.aequivalvis*, *P.humilis*, *P.thurberiana*, *P.rosei*) or 28 (*P.bacillata*).

##### Distribution and ecology.

New World i.e. North, Central, and South America. Found in cold and wet, often high-elevation environments.

##### Notes.

The taxonomic disposition of *Podogrostis* as part of *Agrostis* or a separate genus has long been an obstacle to transfer of the species to the genus. Now that molecular evidence has confirmed the independence of these two genera (Saarela et al. 2017; Konstantin Romaschenko unpublished data) for the type species as well as other species from North America, we feel confident that the genus can be expanded based on shared morphological characteristics. The genus is here considered to contain at least ten distinct species (*Podagrostisaequivalvis*, *P.bacillata*, *P.colombiana*, *P.exserta*, *P.humilis*, *P.liebmannii*, *P.rosei*, *P.sesquiflora*, *P.thurberiana*, *P.trichodes*). Aside from the characters mentioned in the key above, species of *Podagrostis* from Guatemala to NW South America can be easily distinguished from *Agrostis* species with well-developed paleas by the leaf blades being involute or convolute, while being generally flat in the *Agrostis* species with well-developed paleas (*A.capillaris* generally has basal blades involute and culm blades flat). Further distinction of *P.exserta* and *P.trichodes* from other species of *Agrostis* with well-developed paleas in Guatemala and NW South America can be made by the very short panicles, usually < 5 cm long, versus panicles > 5 cm long in the latter.

In high-elevation Guatemala and páramos of Central and NW South America, species are known to only have a densely tufted or tussock-forming habit with intravaginal innovations. A loosely tufted habit and extravaginal innovations, that often leads to a rhizomatous or subrhizomatous habit, is only found in species from Mexico (*P.liebmannii*, *P.rosei*), the USA and Canada (*P.aequivalvis*, *P.thurberiana*) and from Chile and Argentina (*P.sesquiflora*).

*Calamagrostismeridensis* (Luces) Briceño, a species from Venezuelan páramos that is stated to have a rhizomatous habit (Briceño 2010) and which Sylvester et al. (2019b) mentioned may possibly bear affinity to *Podagrostis*, does share certain characteristics with *P.sesquiflora*, such as the flat leaf blades and callus and rachilla apex with short hairs. However, stipitate florets and certain spikelets with a second, albeit reduced, floret at the end of the rachilla prolongation (Briceño 2010; Sylvester pers. observation) suggests this species more likely belongs in *Deschampsia* P. Beauv. The generic placement of *C.meridensis* needs to be confirmed with molecular data.

### Key to the species of *Podagrostis* that are accepted in Latin America

**Table:** 

1	Leaf blades flat; tillers extravaginal, with cataphyllous shoots present; spikelets (1.8–)1.9–2.5 mm long; from Argentina and Chile (*P.sesquiflora*) or Mexico (*P.liebmannii*, *P.rosei*)	**2**
–	Leaf blades involute or convolute, filiform or acicular; tillers intravaginal, cataphyllous shoots absent; spikelets 1–2 mm long, or 2.8–4.2 mm long in *P.colombiana*; from NW South America (*P.colombiana*, *P.trichodes*), Guatemala (*P.exserta*), or Costa Rica and Panama (*P.bacillata*)	**4**
2	Panicle contracted and slender, 2–6.5 × 0.25–1 cm, spikelets present from near the base; callus with short hairs 0.1–0.2 mm long; rachilla extension distinct, 0.2–0.4 mm long, often with short hairs distally; from Argentina and Chile	***P.sesquiflora* (E. Desv.) Parodi ex Nicora**
–	Panicle usually lax and open, less often contracted, 7–18 × (1–)2.5–7 cm, spikelets in the distal 1/3, the lower 2/3 naked; callus glabrous; rachilla extension distinct, 0.3–0.5 mm long, or rudimentary and sometimes absent, glabrous; from Mexico	**3**
3	Lemmas with a well-developed awn inserted in the lower 1/3, straight or geniculate, reaching the apex of the lemma or glumes or briefly surpassing them; leaf blades usually dimorphic, those of the tillers filiform and narrow, 0.2–0.6(–1) mm wide when opened out, flat or folded, those of the culm 1–2.6 mm wide, flat or slightly convolute towards their apices	***P.rosei* (Scribn. & Merr.) Sylvester & Soreng**
–	Lemmas awnless; leaf blades not notably different between tillers and culm, filiform or flat, (0.5–)1–3.5 mm wide	***P.liebmannii* (E. Fourn.) Sylvester & Soreng**
4	Panicle contracted and slender, < 1.2 cm wide, spikelets present from near the base; spikelets 2.8–4.2 mm long; upper glume 3-veined; plants forming small tussocks with a basal mat of leaves 5–26 cm tall	***P.colombiana* Sylvester & Soreng**
–	Panicle lax and open, 1–8 cm wide, spikelets in the distal 1/3, the lower 2/3 naked; spikelets 1–2 mm long; upper glume usually 1-veined, sometimes 3-veined in *P.trichodes*; plants forming short tufts with a basal mat of leaves often < 10 cm tall (to c. 17 cm tall in *P.bacillata*)	**5**
5	Leaf blade abaxial surface finely to densely scabrous; culms usually with at least one elongated internode visible (not including the terminal culm segment below the panicle), with at least 1 node usually visible at flowering; basal mat of leaves usually 6–13 cm tall, to 17 cm tall; leaf blades 2–15 cm long; ligules 1.7–4.3 mm long; panicles 4–11 cm long, panicle branches and pedicels smooth; spikelets 1.7–2 mm long; glume keels scaberulous just in the distal 1/3, surfaces smooth; lemmas smooth; rachilla 0.3–1.4 mm long; from páramos of Costa Rica and Panama	***P.bacillata* (Hack.) Sylvester & Soreng**
–	Leaf blade abaxial surface finely to densely scabrous (*P.trichodes*) or smooth (*P.exserta*); culms usually without visible elongated internodes (NB. this does not refer to the terminal culm segment below the panicle), with 0(–1) nodes visible at flowering; basal mat of leaves usually < 5 cm tall, to 10 cm tall; leaf blades 1–4 cm long, rarely longer, lightly to densely scabrous or smooth; ligules 1–2(–2.5) mm long; panicles 2–5(–6) cm long, panicle branches and pedicels smooth (*P.exserta*) or usually lightly to densely scabrous (*P.trichodes*); spikelets 1–2 mm long; glume keels scabrous just in the distal 1/3 to throughout their length, surfaces smooth or scabrous distally; lemmas smooth (*P.exserta*) or lightly to densely scabrous (*P.trichodes*); rachilla 0.2–0.5 mm long; from various localities, not found in Costa Rica or Panama	**6**
6	Leaf blade abaxial surface moderately to usually densely scabrous; spikelets 1–1.5 mm long; glume keels scabrous just in the distal 1/3 to throughout their length, surfaces smooth or scabrous distally; lemmas moderately to densely scabrous; panicle branches usually lightly scabrous, infrequently smooth; pedicels usually lightly scabrous, rarely smooth; from Colombia and Venezuela to Peru	***P.trichodes* (Kunth) Sylvester & Soreng**
–	Leaf blade abaxial surface smooth; spikelets (1.2–)1.5–2 mm long; glume keels scaberulous just in the distal 1/3, surfaces smooth; lemmas smooth; panicle branches smooth; pedicels smooth; from Guatemala	***P.exserta* (Swallen) Sylvester & Soreng**

#### Podagrostis bacillata

Taxon classificationPlantaePoalesPoaceae

(Hack.) Sylvester & Soreng comb. nov.

4CC1F9F3-2CCA-5761-AB45-65287E2DD1AA

urn:lsid:ipni.org:names:77209699-1

Fig. 1

Agrostis bacillata Hack., Oesterr. Bot. Z. 52(2): 59. 1902.

##### Type.

Costa Rica. Cerro de la Muerte, Jan. 1897, H. Pittier 10477 (***holotype***: W (W19160027240 [image!]); ***isotypes***: BAB (BAB00000206 [image!]), BR (BR0000006595845 [image!]), G (G00192030 [image!]), US (US00131726 [not seen], US00131725 [not seen], US00131136 fragm. [not seen]).

**Figure 1.**
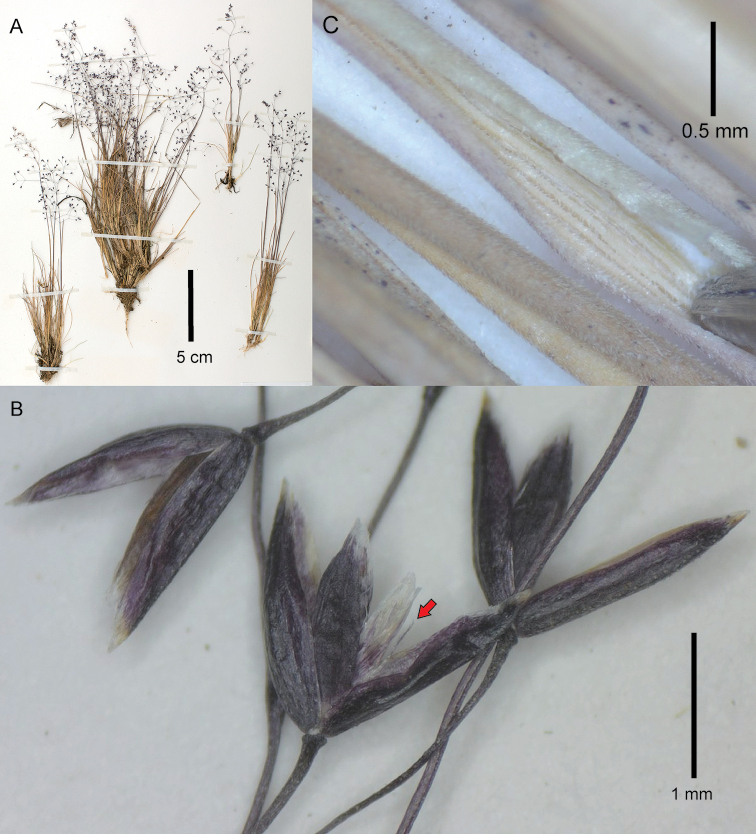
*Podagrostisbacillata*. **A** Whole plant **B** spikelets, the center spikelet at anthesis with the palea opened out and the rachilla prolongation visible (indicated with a red arrow) **C** leaf blades showing the densely scabrous abaxial surface and the central blade opened out to reveal the scabrous adaxial surface. **A, B** Images of specimen A.M. Evans 145 (US2589766A) courtesy of United States National Herbarium (US) **C** image of specimen R.W. Pohl 11705A (US3096988).

##### Description.

***Tuftedperennial*** forming dense tufts, with the basal mats reaching c. 6–17 cm tall and inflorescences usually well-exserted from the basal foliage. ***Tillers*** intravaginal. ***Culms*** 10–30(–50) cm tall, erect, simple, delicate; ***nodes and internodes*** terete, smooth, usually with at least 1 or 2 elongated internodes visible, with (0–)1–3 nodes exposed at flowering, uppermost internode c. 4–13 cm long, longer than the sheath. ***Leaves*** generally basal; ***sheaths*** terete, glabrous, smooth to lightly scabrous with very short scabers; flag sheath 4–13 cm long; basal sheaths 1–4 cm long, striate, becoming fibrous; ***ligules*** 1.7–4.3 mm long, membranous, slightly to usually strongly decurrent with the sheath; upper culm ligules acute to acuminate with a obtuse to acuminate apex, sometimes slightly erose towards the apex; ligules of tillers similar to those of the culm, sometimes slightly shorter; ***blades*** 2–15 cm long, 0.2–0.3(–0.4) mm wide in diameter, involute or convolute, acicular to capillaceous and filiform, straight to slightly curved, abaxial surfaces glabrous, lightly to usually densely scabrous, adaxial surfaces glabrous, lightly to usually densely scabrous on the veins with scabers varying in size from short to long and robust. ***Panicles*** (3.5–)4–11.5 × (1–)3–5 cm, open to slightly congested when young, usually ovoid; ***panicle branches*** ascendant to patent, branched above the middle, filiform, with spikelets not present near the base, glabrous, smooth, longest branches 1–4.5 cm long; ***pedicels*** (1–)2–6 mm long, usually longer than the length of the spikelets, divaricate, glabrous, smooth. ***Spikelets*** 1.7–2 mm long; ***glumes*** remaining on the inflorescence at maturity, equal or subequal, the lower usually slightly longer than the upper, almost equaling the length of the floret to 0.5 mm longer, oblong–lanceolate, slightly keeled, apex broadly acute, glabrous, keels lightly scaberulous just in the distal 1/3, surfaces smooth; lower glume 1- (or 3-)veined, lateral veins usually vestigial; upper glume 1- (or 2- or 3-)veined, lateral veins usually vestigial; ***lemmas*** 1.4–1.6 mm long, glabrous, smooth, faintly 5-veined, apex obtuse, awn lacking or to 0.6 mm long, straight, inserted medially or in the upper 1/3; ***paleas*** well-developed, 0.8–1.5 mm long, usually reaching from ¾ to subequaling the lemma, keels slightly obscure, smooth, apex bifid and sometimes erose; ***rachilla*** 0.3–1.4 mm long, prolonged from the base of the floret, glabrous, rarely smooth, usually scabrous, sometimes with scabers extending into very short hairs < 0.2 mm long. ***Calluses*** 0.1–0.2 mm long, slightly elongated, glabrous, smooth. ***Flowers; lodicules*** c. 0.3–0.5 mm long, lanceolate with acute apices, not lobed; ***anthers*** 3 in number, 0.8–1 mm long. ***Caryopses*** not seen. 2*n* = 28.

##### Distribution and ecology.

Costa Rica and Panamá, páramo grasslands, 2200–3900 m alt. Pohl (1980) mentions that this species apparently flowers throughout the year.

##### Additional specimens examined.

Costa Rica. **Cartago**: Canyon of Rio Tiribi, 3 km NW of Llano Grande, 2200 m, 7 Feb. 1969, R.W. Pohl 11705A (US3096988). **Limon**: Cordillera de Talamanca, Atlantic slope, Kamuk Massif, páramo north-east of the main Kamuk peak, 9°1'–9°17'N, 83°00'–83°0'W, 3000–3300 m alt., 17–18 Sep. 1984, G. Davidse 29277 (US3041608). **San Jose**: Cerro Chirripo, treeless páramo SW of and around summit, 11000–12000 ft [3353–3658 m alt.], 24–26 Aug. 1967, A.M. Evans 145 (US2589766A); Cerro de la Vueltas, páramo, 2700–3000 m alt., 29 Dec. 1925–1 Jan. 1926, P.C. Standley 43625 (US1307153); P.C. Standley 43678 (US1307155); Cordillera de Talamanca, Cerro de la Muerte, Asuncion, 3250 m alt., 13 July 1968, R.W. Pohl 10693 (US3096655).

##### Notes.

The possible affinity of *Agrostisbacillata* to *Podagrostis* was mentioned previously (Pohl and Davidse 1994; Briceño 2010), with Pohl and Davidse (1994) recommending transfer of *A.bacillata* to *Podagrostis*. The species is similar to *P.trichodes* and *P.exserta* (see notes under these taxa on how to differentiate them). The character of adaxial leaf blade surface with conical trichomes intermixed with scabers across a scaberulous surface, mentioned by Pohl and Davidse (1994) to be a key character for differentiating *P.bacillata* from *P.exserta*, was not found in any of the Costa Rican specimens studied at US, including specimen Pohl 10693 that was cited by Pohl and Davidse (1994). All specimens studied of this species were generally densely scabrous on the veins of the adaxial blade surface, with short to long and robust scabers of similar consistency to the abaxial surface. This could be attributed to a difference in interpretation by the authors, although similar long scabers, in a similar density, were found on the adaxial leaf blade surface of specimens of *P.exserta*, meaning this character is not considered useful for species delimitation of these taxa. Instead, we found that a key character for differentiating the two species was the presence or absence of scabrocities on the abaxial leaf blade surface, with *P.bacillata* usually being densely scabrous with short hooks while *P.exserta* is smooth.

Although we have not studied characters of foliar anatomy, Pohl and Davidse (1994) mention these to be important in separation of *P.exserta* and *P.bacillata*. Pohl and Davidse (1994) also mention that spikelets are amply open at maturity in *P.exserta*, while those of *P.bacillata* are more closed, although this distinction is not always clear. Caryopsis shape is also mentioned to be distinct (Pohl and Davidse 1994), although no fruiting specimens were found among specimens at US.

Pohl (1980) mentions the glumes of *P.bacillata* to be 3-veined, although this is not mentioned by Pohl and Davidse (1994) or Morales-Quirós (2003). Most of the spikelets that we examined had 1-veined glumes, with vestigial lateral veins sometimes present.

#### Podagrostis exserta

Taxon classificationPlantaePoalesPoaceae

(Swallen) Sylvester & Soreng comb. nov.

A459F446-99CC-53FB-8BF8-DDFD1C2F21CF

urn:lsid:ipni.org:names:77209700-1

Fig. 2

Agrostis exserta Swallen, Contr. U.S. Natl. Herb. 29(9): 404. 1950.

##### Type.

Guatemala. Dept. Huehuetenango: collected in alpine area, vicinity of Tojquia, Sierra de los Cuchumatanes, 3700 m alt., 5 Aug. 1942, J.A. Steyermark 50119 (***holotype***: US (US00131747 [not seen]); ***isotype***: US (US00131748 [not seen])).

**Figure 2.**
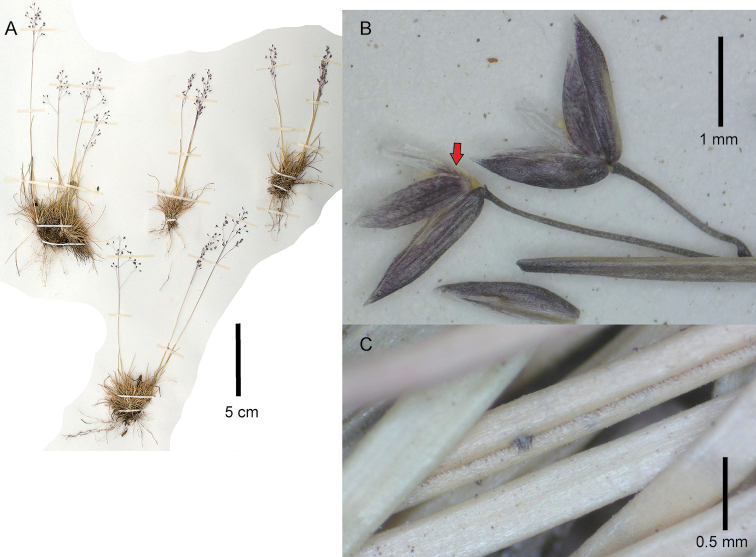
*Podagrostisexserta*. **A** Whole plant **B** Spikelets, the upper glume of the left-hand spikelet has been removed to reveal the rachilla prolongation (indicated with a red arrow) **C** leaf blade adaxial view, showing scabers on the adaxial surface and smooth abaxial surface of the leaf blade. **A** Digitized image of specimen J.H. Beaman 3873 (US01247246) courtesy of United States National Herbarium (US) **B, C** images of specimen G.L. Webster 11826 (US3336153).

##### Description.

***Tuftedperennial*** forming short dense tufts, with the basal mats reaching c. 3–6 cm tall and inflorescences usually well-exserted from the basal foliage. ***Tillers*** intravaginal. ***Culms*** 5–15(–30) cm tall, erect, simple, delicate; ***nodes and internodes*** terete, smooth, nodes usually hidden in the sheaths with 0(–1) nodes exposed at flowering, uppermost internode usually < 1 cm long (to 4 cm long in Steyermark 50216), usually not longer than the sheath. ***Leaves*** generally basal; ***sheaths*** terete, glabrous, smooth; flag sheath 2–5.6 cm long; basal sheaths 0.5–1.5 cm long, striate, becoming fibrous; ***ligules*** 0.8–2(–2.5) mm long, membranaceous, slightly to usually strongly decurrent with the sheath; upper culm ligules acute with a obtuse to truncate apex, usually slightly erose towards the apex; ligules of tillers similar to those of the culm; ***blades*** 1–4(–5.5) cm long, 0.3–0.4(–0.6) mm wide in diameter, involute or convolute, acicular to capillaceous and filiform, usually curved, abaxial surfaces glabrous, smooth, adaxial surfaces glabrous, lightly to usually densely scabrous with scabers usually short, less often long and robust, edges smooth to lightly scabrous with very short hooks. ***Panicles*** (1.2–)2–5 × (0.5–)1–2.5 cm, open to slightly congested when young, usually ovoid; ***panicle branches*** ascendant to patent, branched above the middle, filiform, with spikelets not present near the base, glabrous, smooth, longest branches 0.3–2 cm long; ***pedicels*** (1–)1.5–5 mm long, usually longer than the length of the spikelets, divaricate, glabrous, smooth. ***Spikelets*** (1.2–)1.5–2 mm long; ***glumes*** remaining on the inflorescence at maturity, equal or subequal, the lower usually slightly longer than the upper, subequaling the length of the floret or slightly longer, oblong–lanceolate, slightly keeled, apex broadly acute, glabrous, keels lightly scaberulous just in the distal 1/3, surfaces smooth; lower glume 1- or 3-veined; upper glume 1- or 3-veined; ***lemmas*** (1.1–)1.4–1.6 mm long, glabrous, smooth, faintly to strongly 5-veined, apex obtuse, awn lacking or to 0.5 mm long, straight, inserted medially or in the upper 1/3; ***paleas*** 0.9–1.3 mm long, notable, usually reaching from ¾ to subequaling the lemma, infrequently reaching 2/3 the length of the lemma, keels usually obscure, smooth, apex bifid and erose; ***rachilla*** 0.3–0.5 mm long, prolonged from the base of the floret (sometimes lacking in a small number of spikelets within the inflorescence), glabrous, smooth or scabrous. ***Calluses*** 0.1–0.2 mm long, slightly elongated, glabrous, smooth. ***Flowers; lodicules*** c. 0.5 mm long, lanceolate with acute apices, not lobed; **anthers** 3 in number, 0.7–1.1 mm long. ***Caryopses*** not seen. 2*n*= unknown.

##### Distribution and ecology.

Guatemala, endemic. Grows in alpine grasslands on volcanic soils, 2900–3700 m.

##### Additional specimens examined.

Guatemala. **Huehuetenango**: Sierra de los Cuchumatanes, alpine areas in vicinity of Tunima, 3400–3500 m alt., 7 July 1942, J.A. Steyermark 48321 (US1914763; US2208640); Sierra de los Cuchumatanes, between Tojiah and Chemal at km 319.5 on Ruta Nacional 9N, 3380 m alt., 31 July 1960, J.H. Beaman 3873 (US01247246); Sierra de los Cuchumatanes, between Tojquia and Caxin bluff, summit of Sierra de los Cuchumatanes, 3700 m alt., 6 Aug. 1942, J.A. Steyermark 50216 (US2181354). **Solola**: Woods 11 miles SE of Totonicapan, 3200 m alt., 27 June 1962, G.L. Webster 11826 (US3336153). **Totonicapan**: Desconsuelo, potrero natual, 3100 m alt., Aug. 1954, M. de Koninck 201 (US2153266); On the Tecum Uman Ridge at km 154 on Ruta Nacional No. 1, ca. 20km east of Totonicapan, 3340 m alt., 13 Aug. 1960, J.H. Beaman 4154 (US2381726).

##### Notes.

Similar in general habit to *P.trichodes* (see notes under this species for how to distinguish them). *Podagrostisbacillata* also has smooth panicle branches and pedicels and bears very similar spikelet characteristics to *P.exserta*, with both having spikelets usually measuring > 1.5 mm long, with smooth glumes apart from the keel being lightly scaberulous, and smooth lemmas. *Podagrostisbacillata* can be distinguished from *P.exserta* principally by the culms having at least one visible elongated internode, 4–13 cm long, with usually at least one node exserted from the sheaths (vs. usually no visible elongated internodes or nodes, with the distalmost internode usually < 1 cm long in *P.exserta*), leaf blade abaxial surface lightly to usually densely scabrous (vs. smooth in *P.exserta*), leaf blades usually longer, 2–15 cm long, forming a basal mat reaching c. 6–17 cm tall (vs. usually < 4 cm long, forming a basal mat reaching c. 3–6 cm tall in *P.exserta*), ligules 1.7–4.3 mm long (vs. 0.8–2.5 mm long in *P.exserta*), and a rachilla prolongation 0.3–1.4 mm long (vs. 0.3–0.5 mm long in *P.exserta*), (also see notes under *P.bacillata*). However, one specimen (Steyermark 50216) did have a moderately long internode at c. 4 cm long, as well as slightly longer leaf blades to 5.5 cm long, but could be differentiated based on the short ligules, < 1.4 mm long, short panicles < 3 cm long, and, crucially, the leaf blade abaxial surface being smooth, with scabers only present on the adaxial surface.

The possible affinity of *A.exserta* to *Podagrostis* was mentioned previously (Pohl and Davidse 1994; Briceño 2010), with Pohl and Davidse (1994) recommending transfer of *A.exserta* to *Podagrostis*. Despite repeated efforts by different researchers (e.g. P. Barbera, Y. Herrera, P.M. Peterson, J. R Reichman, K. Romaschenko, L.S. Watrud, pers. comm.) to sequence leaf samples from herbarium specimens of this species, none have been successful to date largely due to the available specimens having mostly aged, brownish leaves. This may be a characteristic of the species in general, with only new fresh shoots being found very early in the growing season and becoming brown at maturity. Successful molecular sampling of this species is necessary.

#### Podagrostis liebmannii

Taxon classificationPlantaePoalesPoaceae

(E. Fourn.) Sylvester & Soreng comb. nov.

1819DFBE-DDA2-5CD8-BEF5-C4583457B9CE

urn:lsid:ipni.org:names:77209701-1

Fig. 3

Agrostis liebmannii (E. Fourn.) Hitchc., N. Amer. Fl. 17(7): 519. 1937. Aperaliebmannii E. Fourn., Mexic. Pl. 2: 97. 1886.

##### Type.

Mexico. [**Veracruz**:] Orizaba, M. Botteri 93 in part (***lectotype***, designated here: P (P00740547 [image!]); isolectotypes: MSC fragm. ex P; ***epitype***, designated here: Mexico. [**Puebla**:] Chinantla, May 1841, F.M. Liebmann s.n. “Plantae mexicanae Liebmann #12591” {handwritten on label} (US (US00595641!)). ***Syntype***: Mexico. [**Puebla**:] Museum Botanicum Hauniense | Plantae mexicanae Liebmann | 1841–43 {printed label}, Gramineae N. 710 | *Aperaliebmannii* Fourn. | [determ.] Fournier | Chinantla. May 1841, {handwritten on label} (NY (NY00345814 [image!]), US [not seen]).

**Figure 3.**
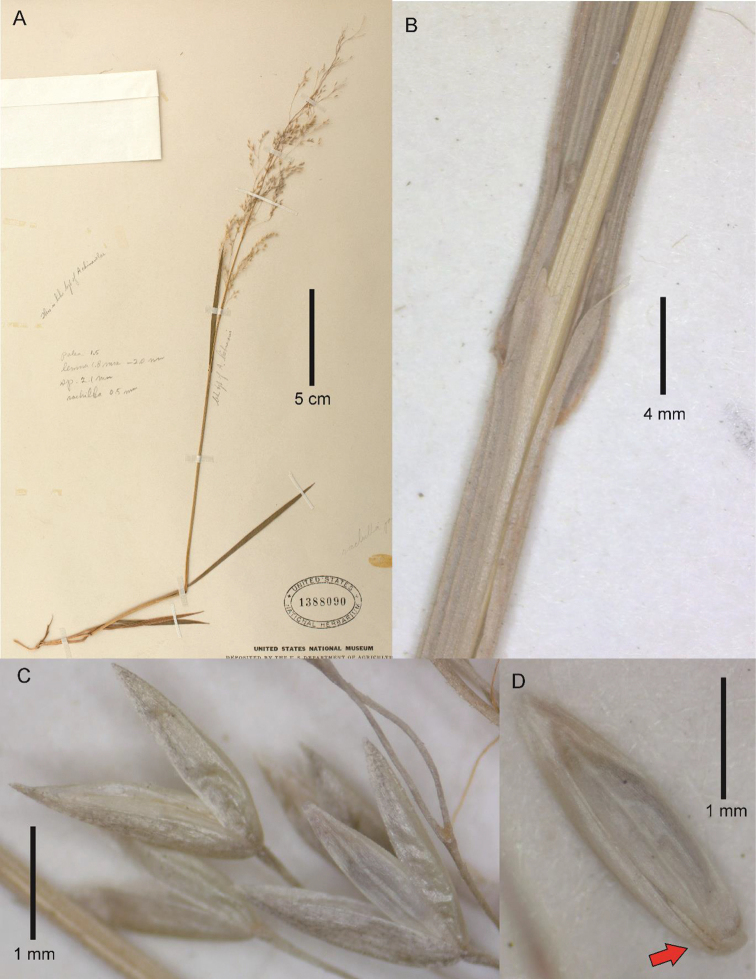
*Podagrostisliebmannii*. **A** Whole plant **B** ligular area of flag leaf **C** section of inflorescence showing spikelets **D** floret, ventral view, with the rachilla prolongation indicated with a red arrow. Images of epitype specimen Liebmann s.n. (US00595641) courtesy of the United States National Herbarium (US).

##### Description.

***Tuftedperennial*** forming lax tufts, with the basal foliage reaching c. 11 cm tall and inflorescences well-exserted from the basal foliage. ***Tillers*** extravaginal. ***Culms*** 35–80 cm tall, erect, simple, delicate; ***nodes and internodes*** terete, nodes smooth, internodes and segment below the panicle smooth throughout (or smooth proximally and lightly scaberulous towards their apices in Moore 3339), usually with at least 1 or 2 elongated internodes visible, with 1–2 nodes exposed at flowering, uppermost internode c. 5.5–11.2 cm long, longer than the sheath. ***Leaves*** basal and cauline; ***sheaths*** terete, glabrous, lower sheaths tending to be smooth, upper sheaths lightly to densely scabrous with short hooks; flag sheath 8–11.5 cm long; basal sheaths 0.5–1 cm long, striate, becoming fibrous, smooth; ***ligules*** c. 0.5–4 mm long, membranaceous or scareous, usually strongly decurrent with the sheath, abaxially scabrous; upper culm ligules 1.5–4 mm long, obtuse to acute, sometimes slightly erose towards the apex; ligules of tillers shorter to those of the culm, c. 0.5–1 mm long, truncate to acute; ***blades*** 2–8.6 cm long, (0.5–)1–3.5 mm wide in diameter, flat, flaccid to firm, basal blades sometimes very narrow, abaxial surfaces glabrous, smooth to lightly scabrous, or usually more densely scabrous further up the culm, adaxial surfaces glabrous, smooth to lightly scabrous on the veins, scaberulous further up the culm, edges scaberulous. ***Panicles*** 7–18 × (1–)2.5–7 cm, open to slightly congested following anthesis, usually ovoid; ***panicle branches*** ascendant to patent, branched above the middle, filiform, with spikelets not present near the base, smooth, longest branches 2–4.7 cm long; ***pedicels*** 1.5–5 mm long, usually longer than the length of the spikelets, divaricate, smooth or lightly scaberulous. ***Spikelets*** (1.8–)2–2.1 mm long; ***glumes*** remaining on the inflorescence at maturity, equal or subequal, the lower usually slightly longer than the upper, subequaling the length of the floret to 0.4 mm longer, lanceolate, slightly keeled, apices acute, glabrous, keels lightly scaberulous just in the distal 1/3, surfaces smooth; lower glume 1- (or 3-)veined, lateral veins, if present, vestigial; upper glume 1- (or 3-)veined, lateral veins, if present, vestigial; ***lemmas*** 1.7–2 mm long, glabrous, smooth, strongly 5-veined with excurrent prominent veins, apex broadly acute, awn absent; ***paleas*** well-developed, 1.4–1.9 mm long, usually reaching from ¾ to subequaling the lemma, keels obscure, smooth, apex bifid and sometimes erose; ***rachilla*** prolonged from the base of the floret, 0.3–0.5 mm long, glabrous, smooth. ***Calluses*** not or slightly elongated, 0.05–0.1 mm long, glabrous, smooth. ***Flowers; lodicules*** c.0.3–0.4 mm long, lanceolate with acuminate apices, not lobed; ***anthers*** 3 in number, 0.6–1 mm long. ***Caryopses*** not seen. 2*n*= unknown.

##### Distribution and ecology.

Mexico, endemic. The authors have only verified specimens from Hidalgo, Puebla and Veracruz states of central Mexico, with Beetle (1983), Dávila et al. (2018), and Sánchez-Ken (2019) mentioning the species to range from Durango state in the north to Oaxaca state in southern Mexico. Found in humid areas of pine and fir forests, *Sphagnum* bogs, and by streams, 2100–2300 m (Beetle 1983).

##### Other specimens examined.

Mexico. **Hidalgo**: Distrito Zacualtipan, pine woods and Sphagnum bogs about 3 miles from Zacualtipan on road to Tianguistengo, 2100 m alt., 4 July 1947, H.E. Moore, Jr. 3339 (US00486609).

##### Notes.

Beetle (1983) appears to consider *Agrostisdurangensis* Mez a synonym of *A.liebmannii*, and states *A.liebmannii* to be distributed as far north as Durango state based on the type locality of *A.durangensis*. However, we consider *A.durangensis* to be a synonym of *A.exarata* Trin. and have only verified specimens from as far north as Hidalgo state. Herrera-Arrieta (2001) cite *A.liebmannii* for Durango state based on specimen Palmer 190 (US00486604), which is treated here as *A.exarata*. Herrera-Arrieta (2014), Dávila et al. (2018) and Sánchez-Ken (2019) also mention *Agrostisliebmannii* to be found as far north as Durango state, but do not include voucher specimens and may have based this on Beetle’s (1983) and Herrera-Arrieta’s (2001) treatments. Certain characteristics in Beetle’s (1983: 82) description of *A.liebmannii* also do not fit the specimens examined, which may be due to the author’s inclusion of *A.durangensis* in the species circumscription. Spikelet size of 2.5 mm mentioned by Beetle (1983: 82) does not fit the specimens studied which had spikelets (1.8–)2–2.1 mm long, although Beetle’s (1983: 73) key to species separates *A.liebmannii* from A.hiemalisvar.laxiflora (Michx.) Beetle (= *Agrostisscabra* Willd.) based on spikelets being c. 2 mm long and shorter than 2.5 mm long. Beetle’s (1983) mention that the tiller blades are more-or-less involute was also not seen, although specimen Moore 3339 (US00486609) was intermixed with another species with involute blades, which might explain this.

*Podagrostisliebmannii* bears close affinity to *P.thurberiana*, a North American species that is found as far south as California (Hitchcock et al. 1969; Harvey 2007). These similarities include a) the overall habit, with tall culms, loosely tufted and subrhizomatous habit with extravaginal shoots; b) spikelet morphology, with spikelets usually < 2.3 mm long, lemmas with excurrent prominent veins and paleas almost subequalling the lemma; c) panicles open and becoming slightly congested following anthesis; d) flat leaf blades. *Podagrostisliebmannii* can be distinguished from the aforementioned species by a) panicles generally much larger, 8–18 × (1–)2.5–7 cm, with patent panicle branches at anthesis (vs. 5–14 × 0.2–3 cm, panicle branches usually ascending at anthesis in *P.thurberiana*); b) callus glabrous (vs. with short hairs to 0.5 mm long emerging from the basal side-ridges of the callus in *P.thurberiana*); c) rachilla glabrous, smooth (vs. short hairs to 0.3 mm long emerging from the apex of the rachilla in *P.thurberiana*).

Fournier (1886: 97) cited two specimens, Liebmann 710 and Botteri 93, in the protologue but only indicated the P herbarium for the Botteri 93 specimen. As we are sure that Fournier saw the Botteri 93 specimen at P, we lectotypify on this collection. Because both the Botteri 93 and Liebmann 710 material included just an inflorescence culm and part of the flag leaf and did not show the basal parts of the plant, we also epitypify the lectotype on Liebmann s.n. (US00595641) that was collected on the same date and at the same locality as the Liebmann 710 collection and includes the basal portion of the plant to help interpret the lectotype. The epitype Liebmann s.n. (US00595641) cited here may in fact be a duplicate of the Liebmann 710 syntype that Fournier (if he ever saw it) did not annotate at the Copenhagen herbarium. The US specimen identified as *Agrostisliebmannii* (“*Apera*” not mentioned) was catalogued as Plantae mexicanae Liebmann #12591; these catalogue numbers are often followed by Liebmann’s field numbers such as the handwritten “710”. The Copenhagen herbarium does not have a Liebmann duplicate bearing either of these numbers, or either of the synonyms.

#### Podagrostis rosei

Taxon classificationPlantaePoalesPoaceae

(Scribn. & Merr.) Sylvester & Soreng comb. nov.

72605C28-A8CE-54D9-B76A-3CBDA76457AB

urn:lsid:ipni.org:names:77209702-1

Fig. 4

Agrostis rosei Scribn. & Merr., Bull. Div. Agrostol., U.S.D.A. 24: 21, f. 5. 1901.

##### Type.

Mexico. **Zacatecas**: Sierra Madre mountains, [between Huasemote, Durango, and San Juan Capistrano], 18 Aug. 1897, J.N. Rose 2373 ***(holotype***: US-301286 [not seen]; isotype: NY (NY00327649 [image!])).

**Figure 4.**
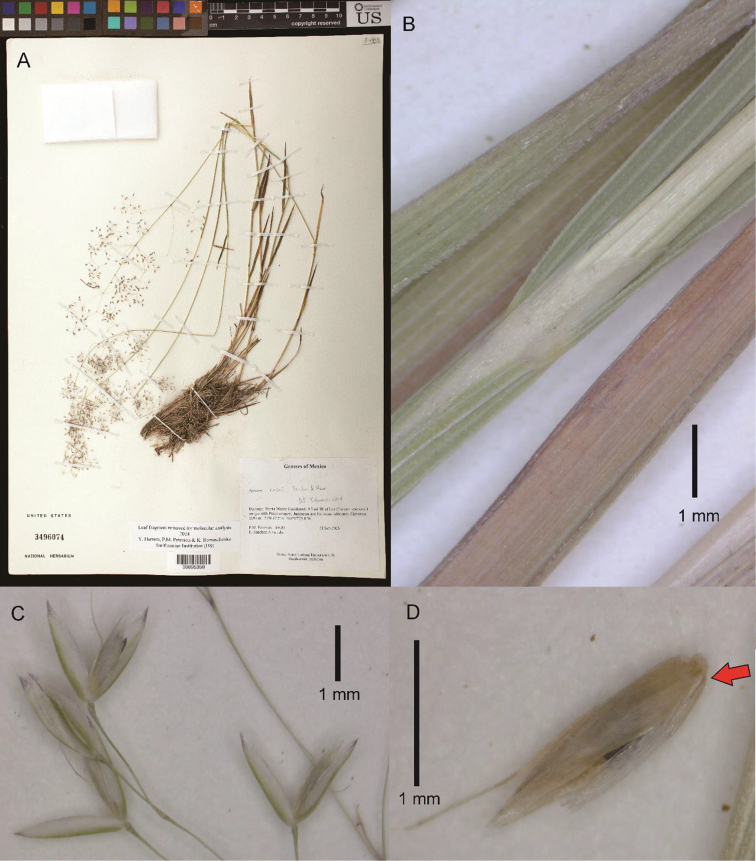
*Podagrostisrosei*. **A** Whole plant **B** ligular area of upper culm leaf **C** section of inflorescence showing spikelets. **D** Floret, lateral view, with the rachilla prolongation indicated with a red arrow. **A** Digitized image of specimen P.M. Peterson 19503 (US00895358) courtesy of United States National Herbarium (US) **B, C** image of specimen P.M. Peterson 19124 (US00900682) **D** image of specimen J.R. Reeder 4662 (US00486613).

##### Description.

***Tuftedperennial*** forming lax tufts, with the basal foliage reaching c. 4–9 cm tall and inflorescences well-exserted from the basal foliage. ***Tillers*** extravaginal. ***Culms*** 34–65 cm tall, erect or geniculate at the base, simple, delicate; ***nodes and internodes*** terete, nodes smooth, internodes and segment below the panicle smooth throughout, usually with at least 2 elongated internodes visible, with 1–3 nodes exposed at flowering, uppermost internode c. 5.6–9 cm long, longer than the sheath. ***Leaves*** basal and cauline, somewhat dimorphic with basal leaf blades filiform, flat or folded, while culm blades are wider and flat; ***sheaths*** terete, glabrous, lower sheaths smooth, upper sheaths smooth to very lightly scaberulous with short hooks; flag sheath 9–11.5 cm long; basal sheaths c. 1–2 cm long, striate, becoming fibrous, smooth; ***ligules*** c. 1–3 mm long, membranaceous to scarious, strongly decurrent with the sheath, sometimes lacerated, abaxially smooth or very lightly scaberulous; upper culm ligules 1.5–3 mm long, obtuse, sometimes deeply lacerated or erose towards the apex; ligules of tillers shorter to those of the culm, c. 1–1.3 mm long, truncate to obtuse; ***blades*** 1.5–9 cm long, 0.2–2.6 mm wide when opened out, basal blades 1.5–7 cm long, 0.2–0.6(–1) mm wide when opened out, usually narrower than the culm blades, filiform, flat or folded, flaccid to slightly firm, culm blades (2.8–)3.5–9 cm long, 1–2.6 mm wide, flat or sometimes slightly convolute towards the apices, flaccid to moderately firm, abaxial and adaxial surfaces glabrous, smooth or usually lightly to moderately scabrous on the veins with short hooks, edges scaberulous to scabrous. ***Panicles*** 8–14 × (1–)2.5–9 cm, open to slightly congested when immature, usually ovoid; ***panicle branches*** ascendant to patent, branched above the middle, filiform, with spikelets not present near the base, smooth, longest branches 2.5–7 cm long; ***pedicels*** 1–2.5 mm long, shorter or longer than the length of the spikelets, divaricate, smooth or lightly scaberulous. ***Spikelets*** 1.9–2.3 mm long; ***glumes*** remaining on the inflorescence at maturity, equal or subequal, the lower usually slightly longer than the upper by up to 0.2 mm, subequaling the length of the floret or to 0.2 mm longer, lanceolate, slightly keeled, apices acute, glabrous, keels completely smooth or scaberulous just in distal 1/3, surfaces smooth; lower glume 1- (or 3-)veined, lateral veins, if present, vestigial; upper glume 1- (or 3-)veined, lateral veins, if present, vestigial; ***lemmas*** 1.6–1.8 mm long, glabrous, smooth, strongly 5-veined with slightly excurrent prominent veins distally, apex broadly acute with 4 deltoid teeth, awn present, 1.6–2 mm long, straight, flexuous or geniculate, inserted in the lower 1/3 of the lemma, sometimes inserted basally c. 0.3 mm from the base, reaching the apex of the lemma, the glumes or sometimes briefly surpassing the glumes, glabrous, smooth proximally, scabrous distally; ***paleas*** well-developed, 1.3–1.7 mm long, usually reaching at least ¾ the length of the lemma to subequaling the lemma apex, keels obscure, smooth, apex bifid and sometimes erose; ***rachilla*** rudimentary or prolonged from the base of the floret, to 0.3 mm long, glabrous, smooth. ***Calluses*** not or slightly elongated, 0.05–0.1 mm long, glabrous, smooth. ***Flowers; lodicules*** c. 0.4–0.5 mm long, lanceolate with acute apices, not lobed; **anthers** 3 in number, 0.8–1 mm long. ***Caryopses*** not seen. 2*n* = 14 (based on Reeder 4662).

##### Distribution and ecology.

Mexico, endemic. Ranges from Durango state in the north to Zacatecas in the south. Found in open forests at high elevations, 2600–2750 m alt. The authors have only verified specimens from Durango and Zacatecas states, with Beetle (1983), Villaseñor Ríos (2016), Dávila et al. (2018) and Sánchez-Ken (2019) variously mentioning the species to also occur in the states of Distrito Federal, Hidalgo, Jalisco, Mexico, Querétaro, and San Luis Potosí.

##### Other specimens examined.

Mexico. **Durango**: Pine forest with scattered oaks and occasional junipers, about 2 miles E of Puerto Buenos Aires, 9000 ft [2743 m alt.], 11 Oct. 1966, J.R. Reeder 4662 (US00486613); Sierra Madre Occidental, 0.5 miles SE of Los Charcos near small arroyo with *Pinuscooperi*, *Juniperus* and *Panicumbulbosum*, 23°0'57.5"N, 104°17'23.0"W, 2690 m alt., 21 Sep. 2005, P.M. Peterson 19053 (US00895358); Sierra Madre Occidental, 2.3 miles E of Los Mimbres along ridgetop with *Pinus*, *Muhlenbergia* and *Quercus*, 23°28'31.4"N, 104°55'3.6"W, 2630 m alt., 25 Sep. 2005, P.M. Peterson 19124 (US00900682).

##### Notes.

This species is distinct from all other species of *Podagrostis* currently circumscribed by the presence of a well-developed awn inserted in the lower dorsal surface of the lemma. Molecular data supports its inclusion in *Podagrostis* (Konstantin Romaschenko, pers. communication) with morphological attributes also corroborating this such as the florets subequalling the apices of the glumes, a well-developed palea > ¾ the length of the lemma, and completely glabrous spikelets. The lax and open, large panicles, and completely glabrous and mostly smooth spikelets, pedicels and panicle branches places it very close to *P.liebmannii*.

#### Podagrostis trichodes

Taxon classificationPlantaePoalesPoaceae

(Kunth) Sylvester & Soreng comb. nov.

49E96924-4034-560C-8452-04696EF4BDB3

urn:lsid:ipni.org:names:77209703-1

Fig. 5

Aira trichodes (Kunth) Spreng., Syst. Veg. [Sprengel]) 1: 276. 1825[1824]. Agrostistrichodes (Kunth) Roem. & Schult., Systema Vegetabilium 2: 361. 1817. Vilfatrichodes Kunth, Nova Genera et Species Plantarum (quarto ed.) 1: 139. 1815[1816].
= Agrostisbogotensis Hack., Repert. Nov. Sp. Fedde 8: 518. 1910. Type: Colombia. S. Cristobal prope Bogota [près de Bogota], [2500–3000 m alt.], 13 July 1908, *F. Apolliniaire* s.n. (holotype: W (W19160027256 [image!]); isotypes: BM (BM000938528 [image!]), MPU (MPU027104 [image!]), SI (SI000495 [image!] fragm. ex US), US (US75365 fragm. [not seen])).

##### Type.

Peru. Crescit in crepidinibus Andium Peruvianum justa Montan, Santa Cruz et Guambos, alt. 1350 hexap. [2469 m alt.], floret Augusto, *F.W.H.A. Humboldt & A.J.A. Bonpland s.n.* (***holotype***: P [not seen]; ***isotypes***: HAL (HAL0106929 [image!]), US (US75364! fragm. ex P)).

**Figure 5.**
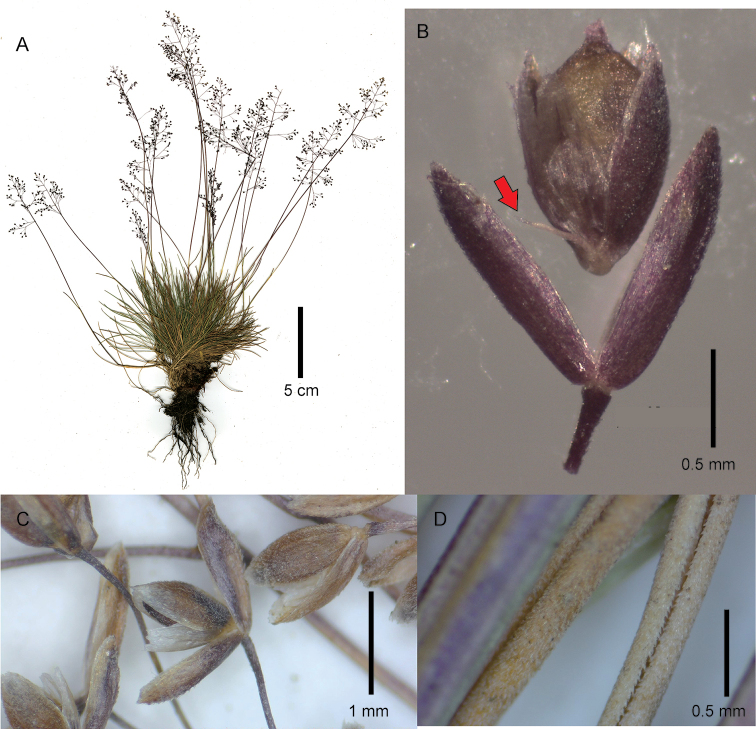
*Podagrostistrichodes*. **A** Whole plant **B** spikelets, with the floret detached and raised above the glumes so that the rachilla prolongation (indicated with a red arrow) can be seen **C** section of inflorescence **D** leaf blade, showing abaxial surface. **A, B** images of specimen L.E. Cuta-Alarcon 362 (FMB) **C, D** images of specimen M.C. Gomez 1 (US3534984).

##### Description.

***Tuftedperennial*** forming short dense tufts, with the basal mats reaching c. 4–11 cm tall and inflorescences well-exserted from the basal foliage. ***Tillers*** intravaginal. ***Culms*** 7–20(–30) cm tall, erect, simple, delicate; ***nodes and internodes*** terete, smooth, nodes usually hidden in the sheaths with 0(–1) nodes exposed at flowering, uppermost internode usually < 1 cm long, usually not longer than the sheath. ***Leaves*** generally basal; ***sheaths*** terete, glabrous, finely to densely scabrous; flag sheath 2–5.6 cm long; basal sheaths 0.7–1.5 cm long, striate, becoming fibrous; ***ligules*** 0.7–1.7(–2.5) mm long, membranaceous, slightly to usually strongly decurrent with the sheath; flag ligules acute with a obtuse to truncate apex, usually slightly erose towards the apex; ligules of tillers 0.7–1.2 mm long, truncate; ***blades*** 1–4 cm long, 0.3–0.4 mm wide in diameter, involute or convolute, acicular to capillaceous and filiform, usually curved, abaxial surface glabrous, finely to densely scabrous, adaxial surface glabrous, lightly to usually densely scabrous with prickle hairs usually short, less often long and robust. ***Panicles*** 2–5(–6) × 1–2(–3) cm, open, ovoid; ***panicle branches*** ascendant to patent, branched above the middle, filiform, with spikelets not present near the base, smooth to usually scaberulous, longest branches 0.8–3 cm long; ***pedicels*** 1–2 mm long, usually longer than the length of the spikelets, divaricate, smooth to usually lightly scabrous. ***Spikelets*** 1–1.5 mm long; ***glumes*** remaining on the inflorescence at maturity or one or both readily caducous at maturity and falling before the floret, equal or subequal, the lower often slightly longer than the upper or less often vice versa, almost equaling the length of the floret or slightly longer, oblong–lanceolate, slightly to distinctly keeled, apex obtuse to acute, glabrous, keels scabrous just in the distal 1/3 to throughout their length, surfaces smooth a scabrous distally; lower glume 1-veined; upper glume 1- or 3-veined; ***lemmas*** 1–1.5 mm long, glabrous, moderately to densely scabrous (‘smooth’ possibly mentioned by Tovar 1993!), sometimes granulose, faintly to strongly 5-veined, apex obtuse, awn lacking or to 0.5 mm long, straight, inserted medially or in the upper half of the lemma; ***paleas*** (0.7–)0.9–1.3 mm long, usually reaching from ¾ to subequaling the lemma, less often reaching 2/3 the length of the lemma, keels obscure to fairly prominent, smooth, apex bifid and erose; ***rachilla*** absent or prolonged from the base of the floret (sometimes lacking in a small number of spikelets within the inflorescence), 0.2–0.5 mm long, glabrous, smooth to scabrous. ***Calluses*** 0.05–0.1 mm long, slightly elongated or not, glabrous. ***Flowers; lodicules*** c. 0.4 mm long, lanceolate with acute apices, not lobed; ***anthers*** 3 in number, 0.4–1 mm long. ***Caryopses*** c. 1 mm long, subterete, sulcus distinct, dark brown with apex dark; hilum 0.25 mm long, narrowly ovoid; endosperm solid. 2n = unknown.

##### Distribution and ecology.

Bolivia?, Colombia, Ecuador?, Peru, Venezuela, 2800–4500 m alt. Relatively humid high-Andean puna grasslands of southern and central Peru and páramo grasslands of Ecuador, Colombia and Venezuela. Tovar (1993) mentions that the species may also occur in Bolivia, presumably in high-elevation cool and humid sites such as the Bolivian Yungas which have been referred to as páramo (García and Beck 2006), although no specimens have been verified by the authors. No specimens at the US herbarium were found from Ecuador after careful searching by the first author, although it is mentioned to occur there (Hitchcock 1927; Tovar 1993; Jørgensen and Ulloa-Ulloa 1994; Jørgensen and León-Yánez 1999; Luteyn 1999). In Colombia, the taxon is known from multiple collections from páramos of the Cordillera Oriental of the Colombian Andes, belonging to Departamentos Cundinamarca, Boyacá, Santander, Santander Norte and Cesar. We present new regional records of the species for Departamentos Santander Norte and Cesar which are not mentioned in the recent checklist (Giraldo-Cañas et al. 2016). Giraldo-Cañas et al. (2016) also cite *Agrostistrichodes* for Departamento Meta, in the southernmost part of the Cordillera Oriental, and Departamento Magdalena, which contains páramos of the Sierra Nevada de Santa Marta, although no specimens have been verified. In Venezuela, the species is found in páramos of the Cordillera de Merida.

Usually found in frequently grazed areas where its short basal tufts of leaves are difficult for grazers to reach. Specimens from Peru appear to be found in humid habitats, with the specimens studied by Tovar (1993) collected from the Abra Malaga of the Cusco region which is relatively humid and receives updrafts of moisture-laden air from the Amazon (Sylvester et al. 2014, 2017). While *P.trichodes* is relatively common in páramos of Colombia and Venezuela, it may be that this species is much rarer further south and, in Peru, belongs to a thin band of humid páramo-like vegetation that extends from the Peruvian Jalca down through southern Peru and into the Bolivian Yungas (Antoine Cleef, pers. communication).

##### Other specimens examined.

Colombia. **Boyacá**: Munic. Chiscas, Vereda Rechiniga, Páramo de la Mesa, área húmeda semiperturbada de Páramo, con *Espeletia*, *Puya*, *Ageratina* e *Hypericum*, 6.59515N, 72.44359W, 3741 m alt., 3 Mar. 2018, S.P. Sylvester 3091 (K, US, FMB); Munic. Chiscas, Páramo de Chacaritas, límites entre páramo y superpáramo, 6.62865N, 72.39440W, 4064 m alt., 4 Mar. 2018, S.P. Sylvester 3103 (K., US); Munic. Chiscas, Páramo el Peñon, borde de bosque de *Polylepis*, 6.60119N, 72.43715W, 3917 m alt., 5 Mar. 2018, S.P. Sylvester 3157 (K, US, FMB, COL, UPTC, SI); Munic. Duitama, Páramo de la Rusia, en la carretera que conduce a la Peña Negra, páramo con rocas expuestas, 5,58389N, 73,053263W, 3970 m alt., 21 Nov. 2017, M. Vorontsova 2218 (K, US, FMB, SI). Munic. Duitama, Páramo de la Rusia, vía que conduce a la Vereda Avendaños, 5.93247N, 73.0798W, 3726 m alt., 4 Oct. 2017, S.P. Sylvester 3038 (K, US, FMB, UPTC); Munic. Duitama, Páramo de Agueros, en la vía que conduce a la vereda Avendaños, Se observa evidencia de fuego y pastoreo, 5.91464N, 73.07114W, 3445W m alt., 28 Oct. 2017, S.P. Sylvester 3067ª (K, US, FMB); Munic. Mongua, Páramo de Oceta, Valle de Laguna Negra, vegetación de pajonal frailejonal con presencia de pastoreo de vacunos, 5.69525N, 72.79133W, 3694 m alt., 29 Nov. 2017, L.E. Cuta-Alarcón 354 (K, US, FMB). **Santander**: Paramo de la Angostura, Vereda El Mortino, 06°57'30"N, 72°43'30"W, 3605 m alt., 17 Nov. 2007, M.C. Gomez 1 (US-3534984). **Santander Norte & Cesar**: Limites entre Santander Norte y Cesar jurisdicciones, Cerro de Oroque, 3700–3900 m alt., 22–27 July 1974, H. Garcia-Barriga 20588 (US29665591; US2966621).

Venezuela. **Mérida**: Sierra Nevada, 9000 ft, 1847, Funck & Schlim 1630 (US); Sierra Nevada de Santo Domingo, between partaderos and Timotes, Paramo de Mucuchies, Pico Aguila, 4118 m alt., 21–26 Nov. 1959, H.G. Barclay 9685 (US3044346); Sierra Nevada de Santo Domingo, Paramo de Mucubaji, alrededores de la Laguna Grande, 3560–3600 m alt., 19 Nov. 1959, H.G. Barclay 9546 (US3096576); Sierra Nevada de Santo Domingo, Paramo Laguna de Mucubaji, carretera Barinas-Merida, 4200 m alt., 15 Nov. 1958, B. Trujillo 4072 (US3652663). **Trujillo**: Munic. Bocono, Laguna Eco to Pico Guarigay (summit), Monumento Natural Teta de Niquitao-Guirigay, summit of Pico Guarigay, 3600–3870 m alt., 16 Sep. 2003, B. Stergios 20450 (US00772686); Munic. Bocono, Laguna Larga, via Laguna Las Parias to Laguna Eco, Paramo de Motumbo, Monumento Natural Teta de Niquitao-Guirigay, 3400–3600 m alt., 15 Sep. 2003, B. Stergios 20420 (US00772685); Along the border with Merida state, 3400 m alt., 14 Sep. 2003, B. Stergios 20315 (US00772683); B. Stergios 20358 (US00772684); Monumento Natural Teta de Niquitao-Guirigay, sector Las Veguitas, 3060–3080 m alt., 20–21 Aug. 2002, L.J. Dorr 9157 (US00728039); Monumento Natural Teta de Niquitao-Guirigay, Paramo Guirigay, 3400–3600 m alt., 2–3 Aug. 2002, B. Stergios 19851 (US00728050).

##### Notes.

Briceño (2010) noted the possible relationship of *Agrostistrichodes* to *Podagrostis* based on the rachilla prolongation. While studying specimens of *A.trichodes* from páramos of Colombia and Venezuela, SPS noted certain characteristics differed from the type collected in Peru, the protologue, and the description in the treatment of grasses of Peru (Tovar 1993). These characteristics, including presence of a rachilla prolongation emerging from the base of the florets, and lemmas sometimes with a short dorsally inserted awn, are also shared by *A.bacillata* and *A.exserta*, and highlight the connection of this species to *Podagrostis*.

The character of awn presence was not noted for this species by Hitchcock (1927) nor Tovar (1993), although Briceño (2010) mentions this for Venezuelan material. While Hitchcock (1927) highlights the rachilla prolongation as a crucial character for distinguishing this species from other *Agrostis*, Tovar (1993) did not mention it. This information is also lacking from the protologues of both *Vilfatrichodes* and *Agrostisbogotensis*. The *Vilfatrichodes* isotype at HAL bears spikelets which lack a rachilla extension, and lemmas that lack awns (Marcus Lehnert and Natalia Tkach, pers. communication). It appears that Oscar Tovar, when preparing his treatment of the grasses of Peru (Tovar 1993), had only seen the US isotype fragment, which lacks florets. His mention that the glumes are ‘glabrous’ (by which Tovar meant glabrous and smooth) raises ambiguity, although most other characters found in the description and illustration match. The flag leaf ligule of the US isotype fragment reached 1.5 mm long, while Tovar (1993) mentions the ligule to measure 2–2.5 mm long, with most material studied from Colombia and Venezuela having flag leaf ligules to 1.7 mm long, with those of the tillers c. 0.5 mm long. Tovar’s (1993) description seems to have been based largely on Tovar and Rivas-Martínez 8076, 8080 from Abra Malaga of the Cusco region of southern Peru, which were not seen by us. The first author visited the Abra Malaga site to conduct extensive field surveys and botanical collecting during different seasons from 2010–2013 (Sylvester et al. 2014, 2017) but no specimens were encountered. Aside from the type, no specimens from Peru have been located despite careful searching through the US herbarium.

*Podagrostistrichodes* closely resembles *P.exserta* and *P.bacillata*, considered endemic to alpine grasslands of Guatemala or páramos of Costa Rica and Panama, respectively (Pohl and Davidse 1994). Key similarities include: a) an overall similar habit (i.e. short tufted herbs with exserted open panicles); b) involute or convolute, acicular or filiform leaf blades; c) presence of a short glabrous rachilla extension emerging from the base of the floret; and d) a short awn often found inserted medially on the lemma dorsal surface. Both *P.bacillata* and *P.exserta* have smooth panicle branches, pedicels, glume surfaces (with only the keels being lightly scaberulous), and lemma surfaces while these are usually lightly to densely scabrous in *P.trichodes*, although specimens have been encountered with almost smooth panicle branches and pedicels [e.g., M.C. Gomez 1 (US3534984), H.G. Barclay 9685 (US3044346), 9546 (US3096576)]. The overall habit of *P.exserta* more closely resembles that of *P.trichodes* than *P.bacillata*, in lacking a visible elongated culm internode and having a shorter panicle (< 5 cm long vs. 4–11 cm long in *P.bacillata*). However, *P.exserta* can be differentiated from *P.trichodes* in having smooth leaf blade abaxial surfaces, lemma surfaces, panicle branches, and pedicels (vs. usually scaberulous to densely scabrous, panicle branches and pedicels infrequently smooth in *P.trichodes*), its glume keels and surfaces being mostly smooth with only few prickle hairs found on the keel distally (vs. glume keels often densely scabrous for most their length with surfaces often scabrous distally in *P.trichodes*), and larger spikelets (usually 1.5–2 mm long vs. 1–1.5 mm long in *P.trichodes*).

*Podagrostisbacillata* can be differentiated from *P.trichodes* in having culms with at least one visible elongated internode and an exserted node (vs. usually without a visible elongated internode and exserted node in *P.trichodes*), panicles usually larger, 4–11 cm long (vs. 2.5–6 cm long in *P.trichodes*), panicle branches and pedicels generally smooth (vs. usually lightly to densely scabrous, infrequently smooth in *P.trichodes*), longer spikelets, 1.7–2 mm long (vs. 1–1.5 mm long in *P.trichodes*), glumes smooth apart from the lightly scaberulous keel (vs. glume keels often densely scabrous for most their length, with surfaces often scabrous distally in *P.trichodes*), lemmas smooth (vs. lightly to densely scabrous in *P.trichodes*), and rachilla prolongation 3–1.4 mm long (vs. 0.2–0.5 mm long in *P.trichodes*).

Specimens from páramos of Departamento Boyacá, Colombia, were noted to have the unusual character of glumes being readily caducous at maturity and falling before the floret, with mature inflorescences lacking glumes and only the florets remaining on the pedicels. It is not clear whether this may be a reaction to a pathogen or whether it is taxonomically informative since other specimens sometimes lack this character.

Certain specimens of Freire Apolliniaire are annotated as isotypes of *Agrostisbogotensis* at P (P00740431 [image!]) and NY (NY00327650 [image!], NY00688633 [image!]) that differ in collection dates, collection numbers, and/or localities from the holotype, with NY00688633 also obviously not the same species. These should be disregarded as type material and reexamined. Apolliniaire s.n. K000308373 may be an isotype but the full collection date is missing to help clarify this.

## Supplementary Material

XML Treatment for Podagrostis

XML Treatment for Podagrostis bacillata

XML Treatment for Podagrostis exserta

XML Treatment for Podagrostis liebmannii

XML Treatment for Podagrostis rosei

XML Treatment for Podagrostis trichodes
